# The effect of cooling procedures on monomer elution from heat-cured polymethyl methacrylate denture base materials

**DOI:** 10.1590/1678-7757-2022-0161

**Published:** 2022-07-22

**Authors:** Nick POLYCHRONAKIS, Maria DIMITRIADI, Gregory POLYZOIS, George ELIADES

**Affiliations:** 1 National and Kapodistrian University of Athens School of Dentistry Department of Prosthodontics Athens Greece National and Kapodistrian University of Athens, School of Dentistry, Department of Prosthodontics, Athens, Greece.; 2 National and Kapodistrian University of Athens School of Dentistry Department of Biomaterials Greece National and Kapodistrian University of Athens, School of Dentistry, Department of Biomaterials, Greece.; 3 National and Kapodistrian University of Athens School of Dentistry Department of Prosthodontics Athens Greece National and Kapodistrian University of Athens, School of Dentistry, Department of Prosthodontics, Athens, Greece.; 4 National and Kapodistrian University of Athens School of Dentistry Department of Biomaterials Greece National and Kapodistrian University of Athens, School of Dentistry, Department of Biomaterials, Greece.

**Keywords:** Heat-cured denture base acrylics, Cooling procedures, Methyl methacrylate monomer elution, Ultra-fast liquid chromatography

## Abstract

**Objective:**

To evaluate the amount of methyl methacrylate (MMA) released in water from heat-cured polymethyl methacrylate (PMMA) denture base materials subjected to different cooling procedures.

**Methodology:**

Disk-shaped specimens (Ø:17 mm, h:2 mm) were fabricated from Paladon 65 (PA), ProBase Hot (PB), Stellon QC-20 (QC) and Vertex Rapid Simplified (VE) denture materials using five different cooling procedures (n=3/procedure): A) Bench-cooling for 10 min and then under running water for 15 min; B) Cooling in water-bath until room temperature; C) Cooling under running water for 15 min; D) Bench-cooling, and E) Bench-cooling for 30 min and under running water for 15 min. A, B, D, E procedures were proposed by the manufacturers, while the C was selected as the fastest one. Control specimens (n=3/material) were fabricated using a long polymerization cycle and bench-cooling. After deflasking, the specimens were ground, polished and stored in individual containers with 10 ml of distilled water for seven days (37^o^C). The amount of water-eluted MMA was measured per container using isocratic ultra-fast liquid chromatography (UFLC). Data were analyzed using Student’s and Welch’s t-test (α=0.05).

**Results:**

MMA values below the lower quantification limit (LoQ=5.9 ppm) were registered in B, C, E (PA); E (PB) and B, D, E (QC) procedures, whereas values below the detection limit (LoD=1.96 ppm) were registered in A, D (PA); A, B, C, D (PB); C, D, E (VE) and in all specimens of the control group. A, B (VE) and A, C (QC) procedures yielded values ranging from 6.4 to 13.2 ppm with insignificant differences in material and procedure factors (p>0.05).

**Conclusions:**

The cooling procedures may affect the monomer elution from denture base materials. The Ε procedure may be considered a universal cooling procedure compared to the ones proposed by the manufacturers, with the lowest residual monomer elution in water.

## Introduction

Heat-cured polymethyl methacrylate (PMMA) is frequently used to fabricate denture bases due to the favorable physical,^1 , 2^ mechanical,^3 , 4^ chemical^5 , 6^ and aesthetic properties^7 , 8^ of the processed material. Nevertheless, the biological properties of PMMA show limitations mostly associated with the presence of residual monomers or their byproducts in the set material.^9 - 11^ These species, such as methyl methacrylate (MMA), dimethacrylate (crosslinking) parent monomers, catalysts or formaldehyde are released after short intraoral exposure periods,^12 , 13^ whereas hydrolytic or biodegradation byproducts are released after long intraoral exposure.^10 , 11 , 14 - 16^

Since MMA is the main eluent from heat-cured PMMA denture base resins, many laboratory methods have been developed to quantify residual MMA monomer in the polymerized materials, such as infrared spectroscopy,^17^ gas chromatography (GC),^18 - 20^ high-performance liquid chromatography (HPLC)^21 - 26^ and ultraviolet spectrophotometry.^27 , 28^ From these methods, the chromatographic analyses offer higher detection and quantification limits.

Many studies have assessed the levels of residual MMA monomer regarding the MMA/PMMA ratio, the curing initiation method, the curing conditions, and the post-polymerization treatments. It has been found that an increased MMA/PMMA ratio leads to an increased amount of residual MMA in the set material,^10^ with heat-cured materials possessing less residual monomer than the self-cured.^16 , 21^ Furthermore, studies show that many procedures can reduce the MMA concentration, such as choosing a curing temperature of 100°C,^5 , 11 , 29^ extending the polymerization time,^29 - 31^ implementing a post-polymerization regime at 55°C for 60 min by exposure to microwave irradiation^24^ or by smearing acrylic resin with a light-cured coating.^32^

Recently, it has been documented that the cooling procedures of the processing flasks affect some mechanical properties of heat-cured denture base PMMA materials,^33^ which may be assigned to post-curing reactions. However, the literature lacks information on the effect of these procedures on the MMA release levels, which may be implicated with ealy biocompatibility issues.

The aim of the study was to evaluate the effects of different cooling procedures instructed by the specific manufacturers on the residual MMA elution of representative heat-cured denture base resin materials. The null hypothesis was that there are statistically insignificant differences in the amount of MMA monomer eluted, despite the cooling procedures used.

## Methodology

**Figure 1:**
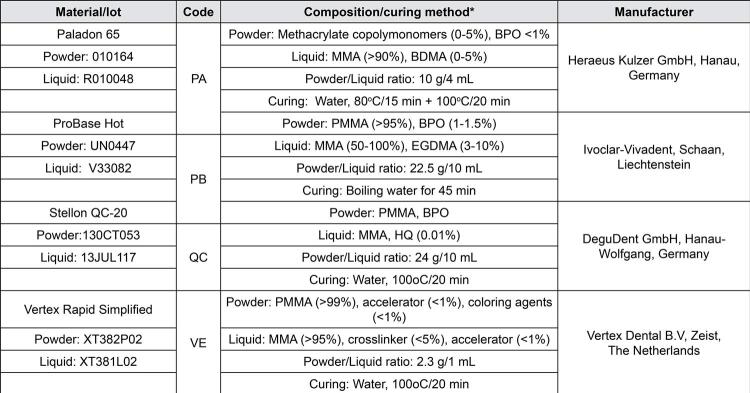
The heat-cured denture base materials used in the study

**Figure 2:**
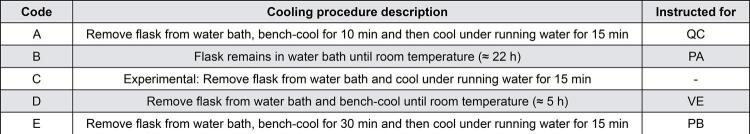
The cooling procedures used in the study

Figure 1 shows the composition, powder/liquid ratios and polymerization methods of the heat-cured denture base materials included in the study. From each material, 15 specimens (17 mm in diameter and 2 mm in thickness) were prepared according to manufacturers’ instructions using a conventional flasking and pressure-pack technique. The discs were divided into five subgroups (n=3, each) depending on the cooling procedures applied ( Figure 2 ). From these procedures, four (A, B, D, E) are recommended by material manufacturers, whereas the fifth one (C: 15 min in cold water) was introduced by the authors as the shortest cooling procedure used.^33^ An additional group (n=3 per material) was fabricated using a generally accepted procedure (polymerization cycle: 74°C for 1.5 h + 1 h at 100°C; cooling procedure: removal from water bath and bench-cooling until room temperature, ≈ 5 h), which served as control.^34^

* According to the manufacturers’ information. MMA: Methyl methacrylate, PMMA: Polymethyl methacrylate, BPO: Benzoyl peroxide, HQ: Hydroquinone, EGDMA: Ethyleneglycol dimethacrylate, BDMA: Tetramethylene dimethacrylate

After deflasking, the PMMA discs were ground in a dry environment using 600 grit SiC papers to remove residual material, conventionally polished with wetted pumice and polishing paste, rinsed with water, air-dried and stored under dark conditions for 24 h (23^o^C/50% RH). Each specimen was then placed in a sealed container with 10 mL distilled water and stored in dark conditions at 37^o^C for seven days. At the end of the storage period, the specimens were removed from the containers, the MMA eluent was subjected to three extractions with 0.6 mL of n-hexane and the final volume of the extract was adjusted to 2.0 mL with n-hexane. The amount of the MMA released was measured using isocratic ultra-fast liquid chromatography (Prominence UFLC system, with LC-20AD solvent delivery unit and SPD-20A UV-Vis detector, Shimadzu, Tokyo, Japan). A reverse-phase column (LiChroCART 250–4 cartridge, LiChrospher 100 RP–8 5 μm column, Merck, Darmstadt, Germany) was used with acetonitrile/water (50:50) mobile phase at 1 ml/min flow rate and detection at 254 nm. Measurements were performed in triplicate per specimen. A three-point calibration curve was used for the quantitative determination of the eluted MMA (5, 10, 50 ppm MMA). The level of quantitation (LoQ) was estimated as 5.90 ppm MMA and the level of detection (LoD) as 1.95 ppm MMA.

## Results

**Figure 3:**
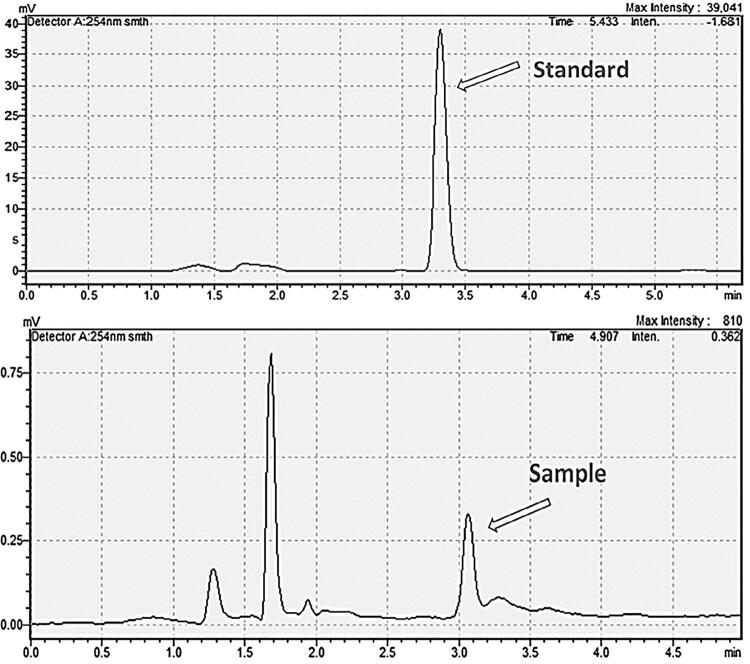
Representative chromatograms of reference MMA (top) and of a specimen elution (bottom)

Figure 3 shows the representative chromatograms of the MMA reference and of a water-eluent specimen. The calibration curve fitted to the linear equation y=1350x-1965.9 (r^2^=0.9997). Table 1 shows the results of the MMA concentration in the water-eluents tested.

**Table 1:** The results of the MMA concentration in the water-eluents of the heat-cured PMMA denture base materials tested*

Cooling procedures	MMA eluted in water (ppm)			
	PA	PB	QC	VE
A	4.6<LoQ	4.6<LoQ	8.5 (1.7)^a,A^	6.4 (0.3)^b,A^
B	<LoD	2.1<LoQ	<LoD	8.7 (2.4)^b^
C	<LoD	4.8<LoQ	13.2 (2.4)^a^	2.9<LoQ
D	2.8<LoQ	2.2<LoQ	<LoD	3.2<LoQ
E	<LoD	<LoD	<LoD	4.2<LoQ
Control	<LoD	<LoD	<LoD	<LoD

* Means and standand deviations (in parentheses).Superscript letters show mean values with insignificant differences within each material group (lower case) and between material groups per treatment (upper case). LoQ: Lower limit of quantitation (5.90 ppm), LoD: Limit of detection (1.95 ppm). Bold characters show the values obtained using the cooling modes suggested by the manufacturers. Data given for results <LoQ represent only mean values

Treatment outcomes below the limit of detection (<LoD) imply that the methodology used failed to discriminate the presence of MMA in material eluents within the concentration range of 0-1.95 ppm (μg/mL). On the other hand, the annotation below the limit of quantitation (<LoQ) indicates that MMA was detectable in the material eluents (>LoD) but could not be accurately quantified within the range of 1.95-5.95 ppm. Thus, the polymerization and cooling conditions of the control group offered better results, despite the type of the material tested. Within each material, results <LoD were limited to the B, C, E (PA); E (PB); B, D, E (QC) procedures, whereas results <LoQ were registered in A, D (PA); A, B, C, D (PB) and C, D, E (VE).

Fully quantitative data were registered only in A, B (VE) and A, C (QC). Student’s t-test showed no statistically significant differences between A and C procedures within the QC group (p=0.652) and for A procedure between QC and VE materials (p=0.827). Welch’s t-test was used for comparison within the VE group (since equal variance test failed- p<0.05), which showed insignificant differences between both cooling procedures (p=0.652).

## Discussion

The results of the present study that there are differences in the amount of detectable water-eluent MMA from some of the heat-cured denture base materials tested, when subjected to different cooling procedures. Therefore, the null hypothesis should be partially rejected.

The experimental design of the study was based on recent findings showing that the cooling procedures proposed by the manufacturers of commercially available heat-cured denture base materials affected some of their mechanical properties obtained under the advised curing mode, such as the Martens hardness, indentation modulus and elastic index.^33^ Since this performance could be associated with post-curing effects during the cooling stage, we question if the different cooling procedures could affect the amount of leachable MMA monomer and, hence, modify the biocompatibility of the set material. Therefore, the amount of labile MMA eluted in water was estimated the amount of labile MMA eluted in water, rather than the total amount of residual MMA in the set products. In the present study the istructed polymerization cycle for each material as a default curing process to address this issue, but the cooling procedures varied; each material was subjected to the different procedures advised by the manufacturers of the four materials tested, including an additional one (C), being the fastest one. A control group was introduced for each material to verify the efficacy of the individual curing and cooling procedures and the cooling regimes, using an accepted curing and cooling methodology.^34^ This would help to understand the relevance of the results obtained, considering that for the materials tested, different curing processes are applicable besides many cooling procedures proposed.

Studies have shown that the HPLC analysis is a suitable method to estimate the residual MMA content in denture base materials^35^ and it has been applied to analyze water-eluted MMA fraction by denture base polymers.^21 , 26 , 32 , 36 , 37^ In the current study an UFLC unit was used an UFLC unit, which offers advantages, such as higher peak resolution, higher signal to noise ratio, faster analysis, increased sensitivity and less consumption of the mobile phase.^38^ The specimen dimensions were similar to previous studies^37^ for comparison purposes. The specimens used were subjected to the standard polishing procedures performed by the dental technicians to better simulate the clinical scenario.

From 72 measurements performed, 33 showed values below the limit of detection (LoD: 1.96 ppm), 27 below the lower limit of quantitation (LoQ: 5.9 ppm) and 12 ranged in mean values from 6.4 to 13.2 ppm of MMA. The LoD is the lowest analyte concentration distinguishable from a blank, usually at 99% confidence level, whereas the LoQ is the lowest analyte concentration that can be reliably detected with repeatability and accuracy.^39^

The results obtained for the control group in all materials imply that the prolonged curing (74°C for 1.5 h + 1 h at 100°C) and cooling (removal from water bath and bench-cooling until room temperature) procedures used were most effective, causing undetectable MMA release in water. From the materials cooled according to the manufacturers’ instructions, PA showed values at the same level of the control; PB and VE caused higher values than the LoD (1.95 ppm) but still below the LoQ (5.9 ppm), and QC reached the value of 8.5 ppm. Based on the same cooling procedures, the differences between the VE and the control reflect the relative advantages of the prolonged curing cycle performed in the control group compared to the one proposed by the VE manufacturer.

The differences found between the cooling procedures within each material group support the hypothesis that the cooling rate may affect the amount of MMA released, although at marginal to the LoQ levels. From the cooling procedures proposed by the manufacturers, the E procedure showed the lowest monomer release in all materials (three materials below the LoD and in one below the LoQ) at a shorter time compared to the D (one material below the LoD and three below the LoQ), which requires more time to reach room temperature. This may imply that a 30 min bench-cooling is mandatory before any other cooling procedure. Additional polymerization of the MMA may occur at the deceleration reaction phase of polymerization during this period of low-cooling rate. A-C procedures showed measurable MMA monomer values in the eluents of QC and VB. These variations may be attributed to differences in material composition (crosslinking monomers, catalyst content, etc) with the effects of the cooling rates.

The results of the present study showed no correlation with any of the mechanical properties of the same materials, as assessed under the same curing and cooling conditions in a previous study.^33^ A possible explanation is that only the fraction of the water-eluted MMA monomer was measured, which may affect the biocompatibility of the denture materials and not the total amount of the remaining MMA monomer in bulk material (pendant groups in the polymer chain), which may affect the mechanical properties of the denture base.^9 , 27 , 37 , 40^ The latter, as specified by the relevant international standard, requires specimen fragmentation, solubilization and total MMA extraction.^35^

Three out of five materials tested showed that the advised cooling procedures caused higher MMA elution than some alternatives. This corroborates the hypothesis that different cooling modalities of heat-cured denture base materials with a given curing cycle affect the amount of MMA monomer release, possibly affecting their biological performance accordingly despite low values measured. Considering the variations in material composition and curing conditions, It may be concluded that cooling procedures involving at least 30 min bench-top storage show the lowest MMA release and can be introduced as a universal cooling procedure for denture base resins. These results, along with the results of a previous study^33^ , show that optimization of the cooling procedures may improve the performance of heat-cured denture base materials, an issue that require further study.

## Conclusions

Under the conditions of this study, the following conclusions can be drawn:

1. From the cooling modes proposed by the manufacturers of the materials tested, bench-cooling for 30 min and placement under running water for 15 min caused minimal residual MMA monomer elution in water when used as a universal procedure for all materials.
2. The cooling procedure instructed for one material showed higher MMA release than all other manufacturers’ proposed procedures applied to the same material.
3. The combination of a long polymerization cycle and bench-cooling to room temperature, such as in the control group, showed MMA values below the limit of detection in all materials tested.
