# ERRATUM

**DOI:** 10.1590/1678-7757-2022er002

**Published:** 2022-05-09

Due to a publishing error the article: “The effect of cooling procedures on monomer elution from heat-cured polymethyl methacrylate denture base materials”, published at Journal of Applied Oral Science 2022;30:e20220161 was printed with the following error:

Where it reads:

**Figure 1:**
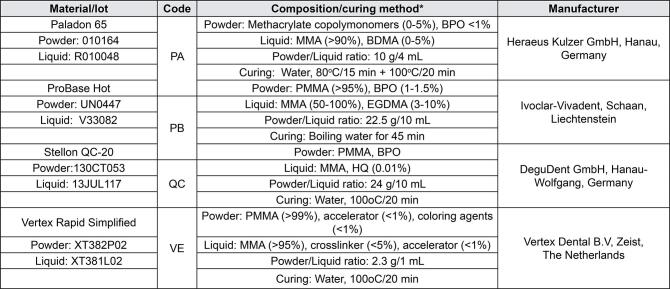
The heat-cured denture base materials used in the study * According to the manufacturers’ information. MMA: Methyl methacrylate, PMMA: Polymethyl methacrylate, BPO: Benzoyl peroxide, HQ: Hydroquinone, EGDMA: Ethyleneglycol dimethacrylate, BDMA: Tetramethylene dimethacrylate

It should read:

**Figure 1:**
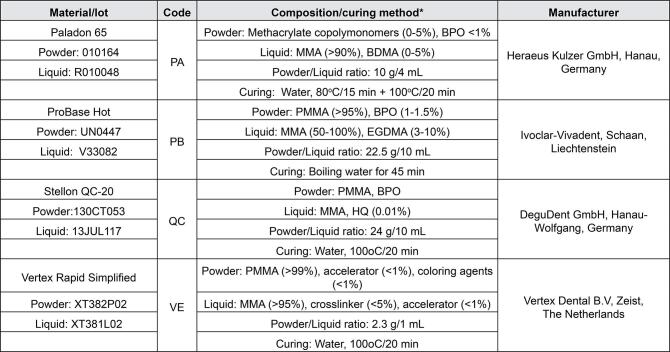
The heat-cured denture base materials used in the study * According to the manufacturers’ information. MMA: Methyl methacrylate, PMMA: Polymethyl methacrylate, BPO: Benzoyl peroxide, HQ: Hydroquinone, EGDMA: Ethyleneglycol dimethacrylate, BDMA: Tetramethylene dimethacrylate

Where it reads:

**Table 1:** The results of the MMA concentration in the water-eluents of the heat-cured PMMA denture base materials tested*

Cooling procedures	MMA eluted in water (ppm)			
	PA	PB	QC	VE
A	4.6<LoQ	4.6<LoQ	8.5 (1.7)^a,A^	6.4 (0.3)^b,A^
B	<LoD	2.1<LoQ	<LoD	8.7 (2.4)^b^
C	<LoD	4.8<LoQ	13.2 (2.4)^a^	2.9<LoQ
D	2.8<LoQ	2.2<LoQ	<LoD	3.2<LoQ
E	<LoD	<LoD	<LoD	4.2<LoQ
Control	<LoD	<LoD	<LoD	<LoD

* Means and standand deviations (in parentheses).Superscript letters show mean values with insignificant differences within each material group (lower case) and between material groups per treatment (upper case). LoQ: Lower limit of quantitation (5.90 ppm), LoD: Limit of detection (1.95 ppm). Bold characters show the values obtained using the cooling modes suggested by the manufacturers. Data given for results <LoQ represent only mean values

It should read:

**Table 1:** The results of the MMA concentration in the water-eluents of the heat-cured PMMA denture base materials tested*

Cooling procedures	MMA eluted in water (ppm)			
	PA	PB	QC	VE
A	4.6<LoQ	4.6<LoQ	**8.5 (1.7)**^a,A^	6.4 (0.3)^b,A^
B	**<LoD**	2.1<LoQ	<LoD	8.7 (2.4)^b^
C	<LoD	4.8<LoQ	13.2 (2.4)^a^	2.9<LoQ
D	2.8<LoQ	2.2<LoQ	<LoD	**3.2<LoQ**
E	<LoD	**<LoD**	<LoD	4.2<LoQ
Control	<LoD	<LoD	<LoD	<LoD

* Means and standand deviations (in parentheses).Superscript letters show mean values with insignificant differences within each material group (lower case) and between material groups per treatment (upper case). LoQ: Lower limit of quantitation (5.90 ppm), LoD: Limit of detection (1.95 ppm). Bold characters show the values obtained using the cooling modes suggested by the manufacturers. Data given for results <LoQ represent only mean values

